# Changes in the oxygen isotope composition of the Bering Sea contribution to the Arctic Ocean are an independent measure of increasing freshwater fluxes through the Bering Strait

**DOI:** 10.1371/journal.pone.0273065

**Published:** 2022-08-25

**Authors:** Lee W. Cooper, Cédric Magen, Jacqueline M. Grebmeier

**Affiliations:** University of Maryland Center for Environmental Science, Solomons, Maryland, United States of America; University of Hyogo, JAPAN

## Abstract

A large volume of freshwater is incorporated in the relatively fresh (salinity ~32–33) Pacific Ocean waters that are transported north through the Bering Strait relative to deep Atlantic salinity in the Arctic Ocean (salinity ~34.8). These freshened waters help maintain the halocline that separates cold Arctic surface waters from warmer Arctic Ocean waters at depth. The stable oxygen isotope composition of the Bering Sea contribution to the upper Arctic Ocean halocline was established as early as the late 1980’s as having a δ^18^O_V_-_SMOW_ value of approximately -1.1‰. More recent data indicates a shift to an isotopic composition that is more depleted in ^18^O (mean δ^18^O value ~-1.5‰). This shift is supported by a data synthesis of >1400 water samples (salinity from 32.5 to 33.5) from the northern Bering and Chukchi seas, from the years 1987–2020, which show significant year-to-year, seasonal and regional variability. This change in the oxygen isotope composition of water in the upper halocline is consistent with observations of added freshwater in the Canada Basin, and mooring-based estimates of increased freshwater inflows through Bering Strait. Here, we use this isotopic time-series as an independent means of estimating freshwater flux changes through the Bering Strait. We employed a simple end-member mixing model that requires that the volume of freshwater (including runoff and other meteoric water, but not sea ice melt) flowing through Bering Strait has increased by ~40% over the past two decades to account for a change in the isotopic composition of the 33.1 salinity water from a δ^18^O value of approximately -1.1‰ to a mean of -1.5‰. This freshwater flux change is comparable with independent published measurements made from mooring arrays in the Bering Strait (freshwater fluxes rising from 2000–2500 km^3^ in 2001 to 3000–3500 km^3^ in 2011).

## Introduction

### 1.1. Significance of freshwater flow through the Bering Strait

Bering Strait is the single largest point source of freshwater to the Arctic Ocean, larger than any river when scaled to deep Arctic Ocean salinity of 34.8 [1]. Freshwater fluxes contributed through the 50 m deep Bering Strait have increased over the past couple decades, based upon mooring measurements [2] and references therein, and these observations are consistent with separate observations of increased freshwater in the Arctic Ocean [3]. Ultimately freshwater balance in Arctic Ocean surface waters and its exchange with the North Atlantic plays a role in affecting the intensity of the Atlantic meridional overturning circulation (e.g., [4,5]). These factors support linkages between the observed increases of freshwater fluxes through Bering Strait and the intensity of the vertical overturning circulation in the north Atlantic. The presence of a cold halocline separating freshened Arctic Ocean surface waters from deeper and warmer Atlantic origin waters in the Arctic Ocean also permits the current presence of seasonal sea ice that would otherwise melt. Finally, water flowing through the Bering Strait has an integrated high nutrient content that promotes high biological productivity in the Bering Strait region [6] and also serves as a marker for Pacific water influence in the Arctic Ocean [7].

**Fig 1:**
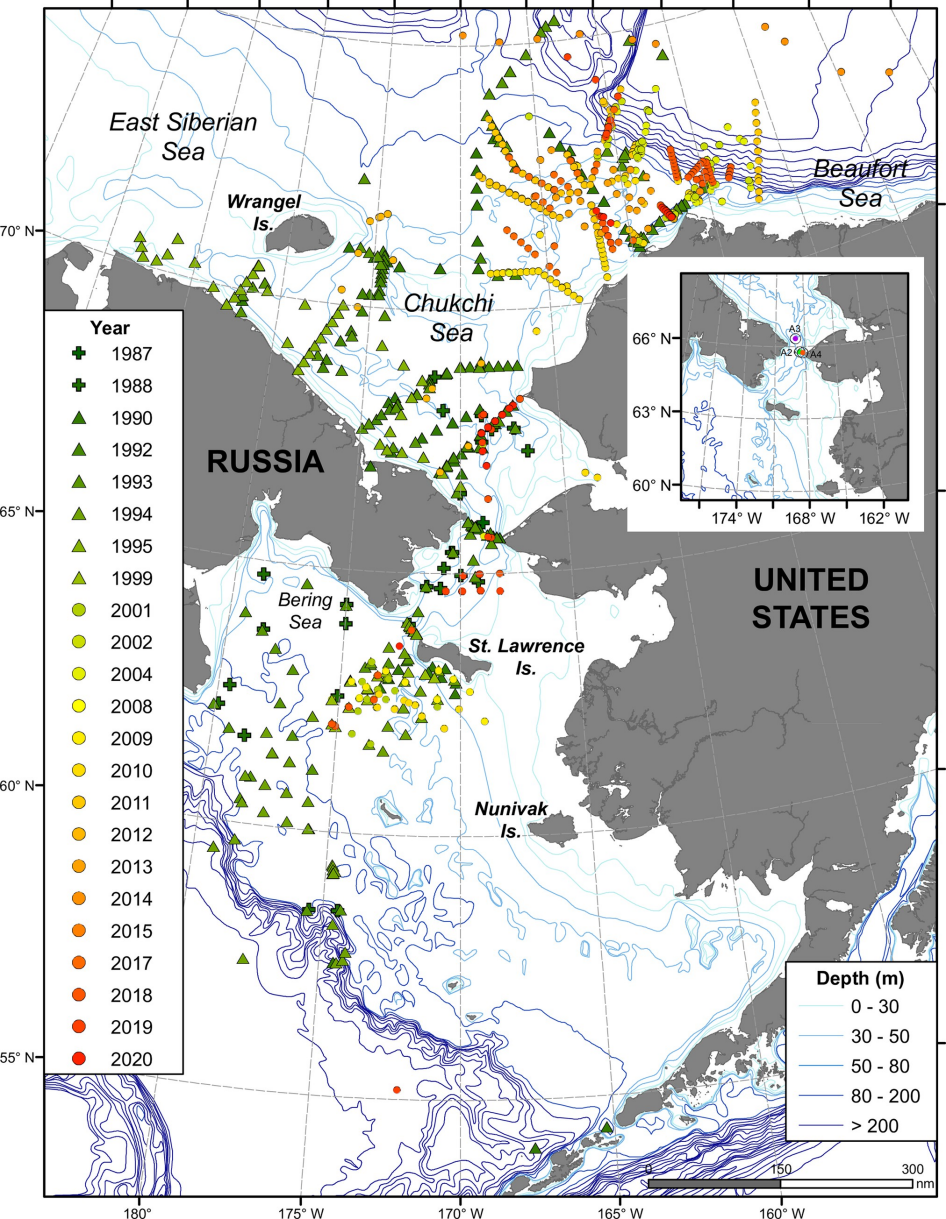
Sampling locations for stable oxygen isotope seawater samples, with salinity between 32.5 and 33.5, collected from 1987 to 2020. Inset shows location of moorings in the Bering Strait, A2, A3 and A4. The figure was made in part with ArcGIS 10.3 under a CC BY license, with permission from ESRI (www.esri.com). The stable oxygen isotope composition associated with nutrient-rich water passing through the Bering Strait however, has the potential to provide an integrated signal that might corroborate the estimates of freshwater flow obtained from moored measurements. This expectation is based upon salinity and δ^18^O values being strongly correlated with each other; both indicate degrees of mixing with fresh water from meteoric sources (snow and rain and subsequent runoff). The high nutrient concentrations in the Bering Strait inflow also provide a basis for further identifying water that has flowed through the Bering Strait.

Despite the importance of determining freshwater fluxes through the Bering Strait, it can be argued that this key parameter is under-monitored. Moorings have not been consistently maintained on the western half of the Strait, and the estimates of current flow and freshwater fluxes for the Strait are based upon extrapolations from just three regularly maintained moorings on the eastern side of the 85 km wide strait (Fig 1, inset). Another challenge for moored observations is the presence of winter sea ice that limits sampling of near surface waters. Bering Strait is sampled reasonably often from shipboard platforms, including for oxygen isotopes (Fig 1), but ship-based under ice sampling is more limited, while the freshwater inventory for much of the year passing through the Strait is influenced by impacts on salinity from brine injection during sea ice formation as well as sea ice melt.

### 1.2. Sea ice formation and variation in oxygen isotopes

While salinity is changed significantly when sea ice forms or melts, δ^18^O values change only to a modest degree as a result of brine injection over the whole water column relative to changes in salinity. When sea ice forms, the isotopic fractionation associated with the phase transition from liquid to solid favors incorporation of the heavier isotope into ice by ~2‰ under equilibrium conditions [8,9], and measurements of melted sea ice cores in the Chukchi Sea are reasonably consistent with that heavy isotope fractionation estimate under field conditions [10]. As a result, if 1 m of sea ice forms over a water column that is 98% liquid water (i.e. a 49 m liquid water column, similar to the depth of the Bering Strait, with an additional 1 m of ice), the brine produced by ice formation is depleted in ^18^O by ~2‰ relative to the ice that is formed. For an ideal, uniform 49 m water column with a δ^18^O value = -1.0‰, a brine contribution from the 1 m surface ice that is formed would have a δ^18^O value = -3.0‰, which when evenly mixed over the whole water column results in an overall water column δ^18^O = -1.04‰, based upon:

Water depth x δ^18^O for each increment, integrated and divided by the total water column,

(49 *-1.0‰) + (1* -3‰) = (-49–3)/50 = -1.04‰ (change in isotopic composition from -1.0‰)

**Fig 2:**
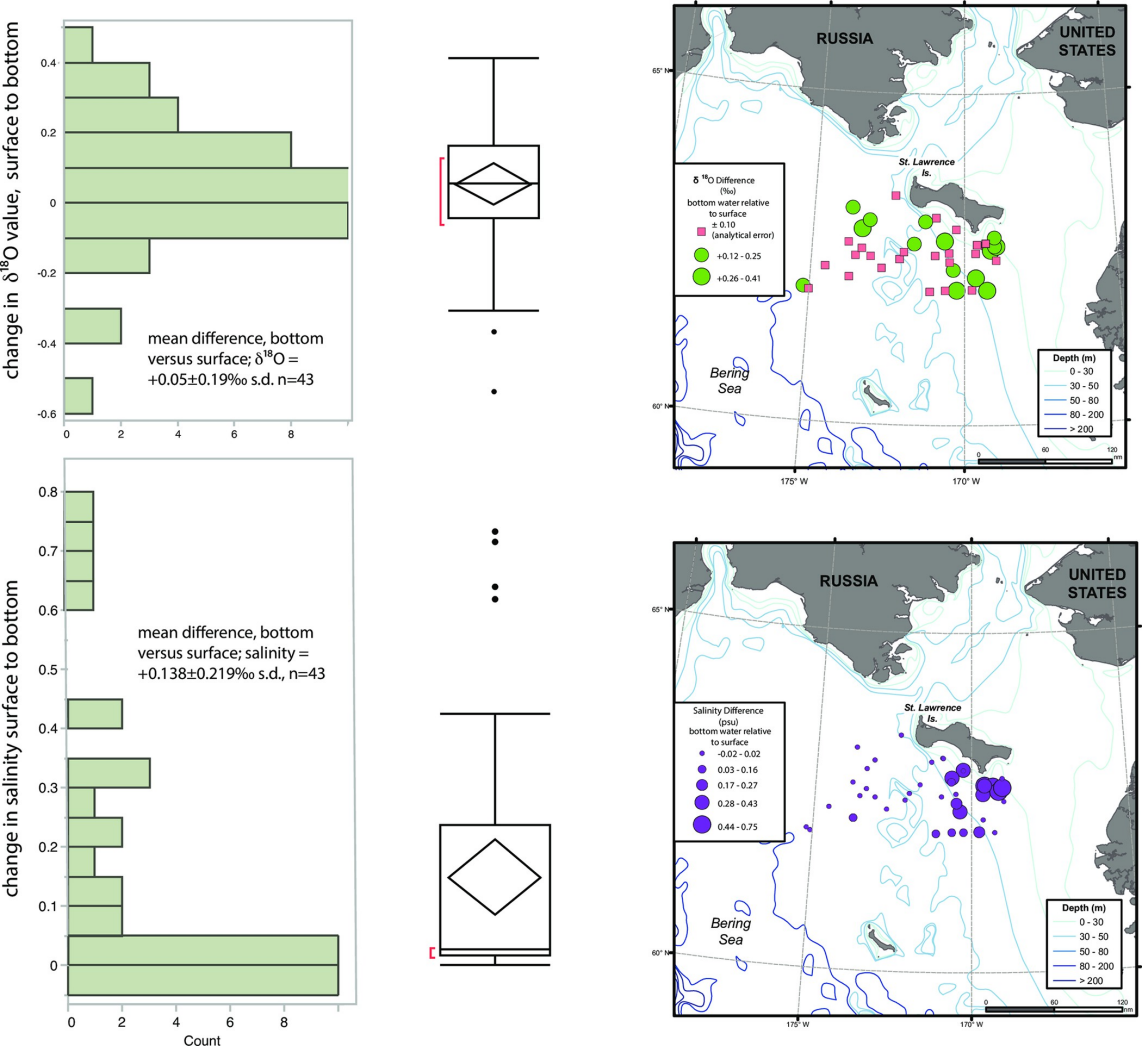
Difference in δ^18^O values (top) and salinity (bottom) between surface waters and bottom (~50 m) in an active area of sea ice formation, St. Lawrence Island polynya, March 2009. Outlier box plot shows 95% confidence limits, n = 43. The figure was made in part with ArcGIS 10.3 under a CC BY license, with permission from ESRI (www.esri.com).

In an ideal, well-mixed 50 m water column, this small change in the isotopic composition results in a depletion of heavy isotope composition of -0.04‰ between sea ice that is forming and underlying water, which is about the same, or smaller than the analytical error associated with the oxygen isotope measurement. In practice, the oxygen isotope variation between surface and bottom water layers on these continental shelves probably is often larger, due to ubiquitous lateral transport [11]; data from a March 2009 cruise of the USCGC Healy during active sea formation [12] shows that the difference in oxygen isotope ratios between surface and bottom waters was smaller than 0.1‰ at 20 of 43 stations; larger changes were however observed elsewhere, but instead of leading to slightly more negative δ^18^O values, for the most part, δ^18^O values were higher, as were salinities (Fig 2), indicating lateral transport of brine. Salinity variation in surface waters in the winter is not constant, but is much less variable spatially [12–14] than in the summer [15]. Moreover, the salinity of water at the time of initial sea ice formation and brine injection (in the Bering Sea) is not critical because additional brine is added during transport north, and our interest is in the blended oxygen isotope composition of water that reaches the depth of the upper Arctic Ocean halocline with its salinity of ~33.1.

### 1.3. Relationship between salinity and δ^18^O value and the available data set

The δ^18^O value of the upper Arctic Ocean halocline was estimated to be ~-1.1 ‰ in the late 1980s [16,17]. The salinity associated with this oxygen isotope composition is 33.1 and water of this salinity, together with a high nutrient content is consistent with Pacific Ocean waters flowing through the Bering Strait [15].

In water samples from the Bering Strait region, and where those water samples have δ^18^O values of ~ -1.1‰ (equivalent to the upper Arctic halocline), salinities range from 31.5 (containing melted sea ice) to 33 (containing brine; [18]). However, Chukchi shelf observations from May-June 2002, prior to significant freshwater contributions from ice melt, [19] indicates that over-winter brine injection dominates on the Chukchi shelf and is described by an apparent mixing line,

$$\delta^{18}O=0.66*\;salinity\;‐23.22‰,\;r^{2}=0.89,\;n=160$$ (1)

If the salinity of the upper Arctic Ocean halocline, 33.1, is entered into this equation, it results in an estimate of the oxygen isotope composition of this water as being -1.37‰ in 2002, compared to the estimate of -1.1‰ of [16]. This difference is larger than the analytical error of the measurement (~0.1‰) and implies a small depletion in the heavy oxygen isotope composition of the upper Arctic halocline between the 1980s and the early 2000s.

Despite these salinity variations introduced seasonally by brine injection, which predominates on the Chukchi shelf, to the south in the Bering Strait itself, the relationship between salinity and δ^18^O values is described by a linear regression [18] that includes a more realistic and less heavy isotope depleted freshwater end-member than in Eq (1) [18],

$$\delta^{18}O=0.57*\;salinity\;‐19.30‰r^{2}=0.81,\;n=231.$$ (2)

It is worth noting however, that this regression includes spring sampling during sea ice melt, so the slope is lower and the freshwater end-member (δ^18^O = -19.30‰) is likely not quite negative enough (compare with flow-weighted runoff from the Yukon River, for example: -20.2‰ [20]). In order to address the isotopic composition of meteoric water likely to contribute to the upper Arctic halocline, it is most realistic therefore to use a regression of a subset of these samples collected only in late summer when there is no longer melted sea ice present in the Bering Strait and sea ice formation is not occurring either. These samples showed a high correlation between δ^18^O values and salinity as well as showing good fidelity to the expected freshwater end-member [18]:

$$\delta^{18}O=0.59*\;salinity\;‐20.0‰r^{2}=0.97,\;n=27$$ (3)

We used this observed regression (3) as a starting point to retrospectively study the range of δ^18^O values that have been observed in seawater samples collected on both the northern Bering and Chukchi shelves in addition to more characteristic upper halocline waters in the Arctic Ocean proper. In this study, we took advantage of this large available data set for the oxygen isotope composition of seawater (Fig 1) that has been collected from 1987 to 2020 in both the northern Bering and Chukchi seas, mostly in the summer, and in waters deeper than 30 m, but also including samples collected as early as March and as late as October. Due to the predominant northward transport of waters into the Arctic Ocean through Bering Strait, it is reasonable to assume that most of these waters sampled (Fig 1) would have reached the Arctic Ocean with the distinguishing characteristics of the Bering Sea input [16]. However, waters flowing through the Bering Strait in ice-free months typically are not saline enough to match the salinity of the upper halocline of the Arctic Ocean (salinity = 33.1). As these waters pass over the shelf during periods of sea ice formation, salinity is increased as described above, and Bering Sea waters with high nutrient content settle out in the upper halocline at a salinity of 33.1.

### 1.4. Assumptions of the approach

The freshwater end-member of meteoric water (freshwater that originates from melted sea ice is treated as non-meteoric) represents the integrated δ^18^O value of precipitation falling near and far field, as well as runoff. The isotopic content is controlled by global patterns of evaporation and condensation as influenced by air temperature, humidity, elevation, water vapor history and other factors [21]. We assume that the oxygen isotope composition of the overall precipitation and runoff making up the freshwater component of the Bering Strait inflow has not changed since the late 1980’s. This assumption is conservative because if there was any decadal scale isotopic variability in precipitation, it would be apparent in other data sources such as the Global Network of Isotopes in Precipitation or more specific Arctic databases such as the Arctic Great Rivers Observatory [22]. This assumption that the oxygen isotope composition of meteoric water has not changed, suggests the possibility of using any changes in δ^18^O values in the upper halocline of the Arctic Ocean as a potentially independent way to estimate changes in the freshwater inflow to the Arctic Ocean through Bering Strait. In other words, if the isotopic composition of precipitation has not changed, any shift from expectation is likely due to a change in the freshwater flux and follows from the direct correlation between freshwater proportions and the δ^18^O value of any seawater mass.

A second, implicit assumption is that the salinity (33.1) of the upper halocline of the Arctic Ocean has not changed over this same time period. Again, this salinity in the North American basins of the Arctic Ocean is associated with a nutrient maximum that is characteristic of the high nutrient waters flowing through the Bering Strait [15], as well as regenerated nutrients from shelf sediments [23]. To the best of our knowledge the nutrient maximum in the upper Arctic Ocean halocline remains associated with a salinity of 33.1, although it has been recently suggested that winter waters transiting Bering Strait are now too fresh for winter-origin, nutrient-rich water to attain that salinity [24]. On the other hand, it is possible that additional brine injection on the Chukchi shelf may still provide for the maintenance of high-nutrient waters in the upper halocline, but the Woodgate and Peralta-Ferriz [24] findings are nevertheless significant and indicate the need for additional measurements of the upper halocline and its nutrient and tracer content in the coming years. The mooring measurements in Bering Strait show that for most of the year water flowing through the Strait has a lower salinity [2] and references therein), but brine injection occurs during ice formation north of Bering Strait, particularly on the Chukchi continental shelf, and this denser water, at least until recently, then settles out at the upper Arctic Ocean halocline at depths of 100–200 m once it is off the shelf in the deeper Arctic Ocean. We discuss in the methodology to follow the expected oxygen isotope composition of these waters, the freshwater content, and follow with a description of our synthesis of approximately 1400 δ^18^O values for seawater with a salinity range of 32.5 to 33.5 that were collected in 23 years from 1987 to 2020. This salinity criteria generally limits the geographical distribution of samples to the northwestern portion of the Bering Sea and the northeastern Chukchi Sea. Waters to the east of St. Lawrence Island in the Bering Sea are typically too fresh to meet the criteria, and sampling in the western Chukchi Sea was relatively limited because of the international border between the United States and the Russian Federation.

## Methods

### 2.1. Empirical considerations

We use empirical estimates of freshwater end-members as a starting point to evaluate how freshwater volumes have changed over the past three decades. The freshwater end-member at zero salinity (δ^18^O = ~-19‰) that is estimated from regression Eq (2), as discussed in the introduction is likely not sufficiently negative because the regression incorporates some water samples that included freshwater fractions originating from melted sea ice [15]. Using inferences such as the flow-weighted oxygen isotope composition of the Yukon River (-20.2; [20], and observations on the Chukchi slope and adjoining deep Canada Basin in 2002 [19] a mixing line between Atlantic water (salinity = 34.8, δ^18^O = +0.3) and meteoric water (salinity = 0, δ^18^O = ~ -20.0) can be expected in the Arctic Ocean [19], but brine injection tends to decrease the δ^18^O value of the apparent freshwater end-member and increase the slope of the relationship [19]. Another less complex approach is to construct a simple mixing line between the expected upper Arctic halocline salinity (33.1) and δ^18^O value (-1.1‰) and the freshwater meteoric end-member (0 salinity) and a δ^18^O value for that end-member = -~20‰,

$$\delta^{18}O=Salinity\;*\;0.604–freshwater\;end‐member$$ (4)

An iterative end-member substitution approach can be used to determine that a freshwater meteoric end-member of -21.1‰ provides the best fit to match a salinity of 33.1 with a δ^18^O of -1.1‰,

$$\delta^{18}O=Salinity\;*\;0.60–21.1‰$$ (5)

This regression, which incorporates Atlantic water present below the upper halocline differs slightly from Eq (3), δ^18^O = Salinity * 0.59–20.0‰, which is based upon data collected in the Bering Strait, where no Atlantic water is present, thus the difference is largely attributable to the presence of a Pacific Ocean end-member (salinity = 34.7, δ^18^O = -0.17) [25], in the Bering Strait samples instead of an Atlantic end-member (salinity = 34.8, δ^18^O = +0.3) [17]. This Pacific end-member based regression has a slightly higher slope for the regression and a more negative δ^18^O value for the estimated freshwater end-member, corresponding to a slightly less heavy-isotope depleted end-member from sources such as the Yukon River (δ^18^O = -20.3‰) [20]. Consequently, we used Eq (3) as a starting point to determine whether observed δ^18^O values in waters “upstream” of the Arctic upper halocline differed significantly from expected values for salinities between 32.5 and 33.5. Eq (5) would by contrast be appropriate once Bering Sea water reaches the depth of the upper Arctic halocline, but again Eq (3) is more appropriate on the shelf. This salinity range of 32.5 to 33.5 was chosen to match those water parcels that were likely to contribute ultimately to the upper Arctic Ocean halocline (salinity = ~33.1) once influenced by additional brine injection over the northern Bering and Chukchi shelves; shifts from expectations can then be attributed to a change in the freshwater flux. An important point is that fresher sources of water such as the Alaska Coastal Water are present in both the Bering and Chukchi seas during the summer when runoff ensues, but these waters are nutrient poor [15] and not present in the winter [12]. Thus, they are not likely to directly contribute to the upper Arctic Ocean halocline through brine injection over winter. While these fresher surface waters ultimately are incorporated into polar mixed waters of the Arctic Ocean, we considered that data to be outside of the scope of our efforts to directly assess the contributions to the upper Arctic halocline, given lower salinity and low nutrient content.

### 2.2. Sample origin and synthesis

Seawater samples were collected, starting in 1987 and in most years up to 2020 from locations in the Bering and Chukchi seas and the deeper Arctic Ocean (Fig 1). Many of these results have been previously presented in other publications [12,15,19] and are also available in data archives, including https://arcticdata.io; http://pacmars.eol.ucar.edu, https://data.giss.nasa.gov/o18data/. Data collected since 2017 have not been previously published and are being archived at http://arcticdata.io. Almost all samples were measured on one of three separate stable isotope mass spectrometers, a VG Instruments dual inlet SIRA II at Oak Ridge National Laboratory, using an offline equilibration of samples with carbon dioxide [26], a ThermoFisher Delta XP at the University of Tennessee, and a ThermoFisher Delta Plus at the Chesapeake Biological Laboratory, both run in a continuous flow mode, and using a ThermoFisher Gasbench peripheral to equilibrate the water samples with carbon dioxide added as a custom mixture to high purity helium. Precision (±0.1‰ or better) was assessed by repeated measurements of internal laboratory working standards, some of which were shared across all three instruments used; these internal standards had been calibrated against international standards (SMOW, GISP, and SLAP); final data was normalized based upon instrument performance with standards [27]. All δ^18^O values are referenced to the Vienna-Standard Mean Ocean Water standard and the statistical package JMP Pro 15.2.0 (SAS Institute) was used for all statistical analyses. A significance criteria of p<0.05 was used to judge the outcomes of statistical tests performed.

### 2.3 Data set criteria used

In developing criteria for waters likely to contribute to the upper Arctic Ocean halocline, we assumed that waters with a minimum salinity of 32.5 from the north Bering Sea into the Chukchi Sea, will receive additional brine injection as the waters pass over the shelf seasonally and could be expected to settle out at or near the upper halocline after transport off the shelf. We also assume that brine injection will become an increasingly more important factor in determining salinity in a northward direction as water flows across the Bering and Chukchi shelves. We excluded all seawater samples with salinity <32.5 because it was less certain that these water samples would have enough brine added to contribute to the upper Arctic Ocean halocline. Furthermore, since fresher waters on the Bering and Chukchi shelves are not associated with high nutrient content [15], it was less likely that these waters would have enough brine injected to reach the upper halocline of the Arctic Ocean and/or also contain sufficient nutrient concentrations. An upper salinity maximum of 33.5 was also set to exclude waters that could potentially settle out below the upper Arctic Ocean halocline. Water samples meeting these criteria (>32.5 salinity <33.5) totaled 1405, of which 389 were collected in the Bering Sea or in Bering Strait, and 1016 samples were collected north of Bering Strait. Thus, these water samples can be categorized as falling into the following categories: 1) collected during periods when ice formation is occurring or 2) are from the depth of the Arctic Ocean halocline, or 3) are classified as Bering Winter Water and collected from near the Chukchi seafloor if still on the shelf [15], so all of these samples are waters that have been salinized over the winter as a result of brine injection. A few additional samples are from the deeper Bering Sea (>~175 m) and have salinities high enough to meet this criterion. The mean salinity of samples collected in Bering Strait or south of the Strait and used in this study was 32.81 ±0.20 SD and for samples collected north of the Strait used in this study, mean salinity was nearly identical, 32.85 ±0.24 SD. By contrast, the mean δ^18^O value for the 389 Bering Sea samples used was -0.94‰± 0.39 SD while the mean δ^18^O value for the 1016 samples collected and used in the Chukchi Sea was -1.27‰ ± 0.39 SD. This difference in isotopic composition, but given similar mean salinity, implies that on average, cumulatively more brine had been injected into the water column in the Chukchi Sea than in the Bering Sea, and confirms the assumption of increasing brine injection influence to the north.

We used the mixing line [Eq (3)] established for waters flowing through the Bering Strait and ultimately off the Chukchi shelf after melted sea ice was no longer present, for predicting the oxygen isotope composition of samples from salinity. We applied this equation to the 1405 water samples that had been collected between 1987 and 2020 with salinities between 32.5 and 33.5. These estimates of stable oxygen isotope composition were then compared to measured δ^18^O values to assess whether there were statistically significant differences between projected and measured δ^18^O values. Given the observations from moorings that freshwater fluxes have increased through the Bering Strait in the past couple decades [2], we hypothesize that δ^18^O values in the salinity range 32.5 to 33.5 should be more negative than estimated from the expected mixing line Eq (4). This would be consistent with decreases in salinity over the time period of study, i.e. a higher proportion of freshwater, meaning more negative δ^18^O values for the seawater sampled.

## Results

**Fig 3:**
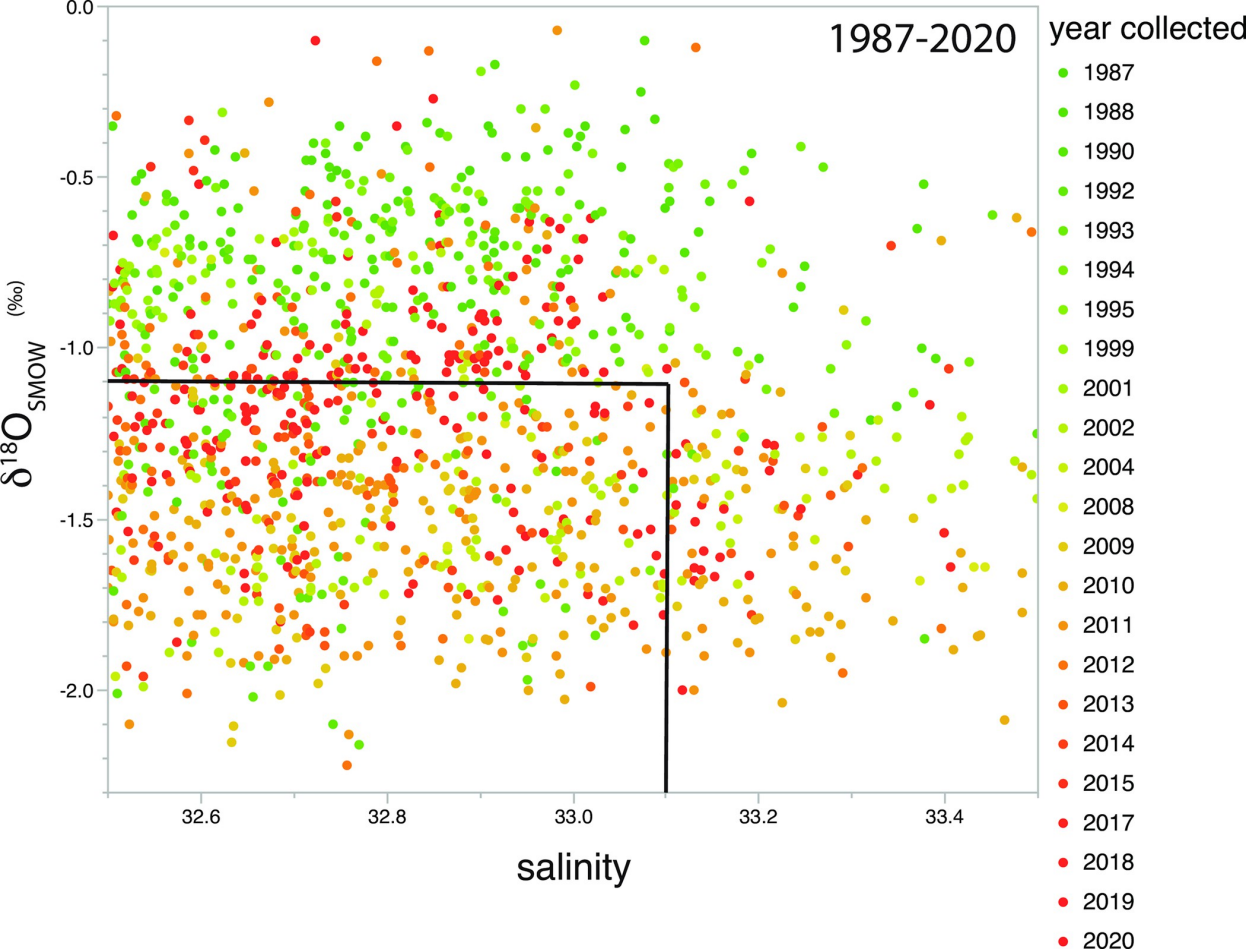
Distribution of δ^18^O values relative to salinity for 1405 samples collected between 1987 and 2020 in the north Bering and Chukchi seas, and Arctic Ocean. The salinity (33.1) and δ^18^O value (-1.1‰) associated with the upper Arctic Ocean halocline are denoted by the two black lines. Color coding of δ^18^O values by years range from greenish for early years (1987–2000), yellowish for middle years (2001–2011), and reddish for recent years (2012–2020) of the time series.

**Fig 4:**
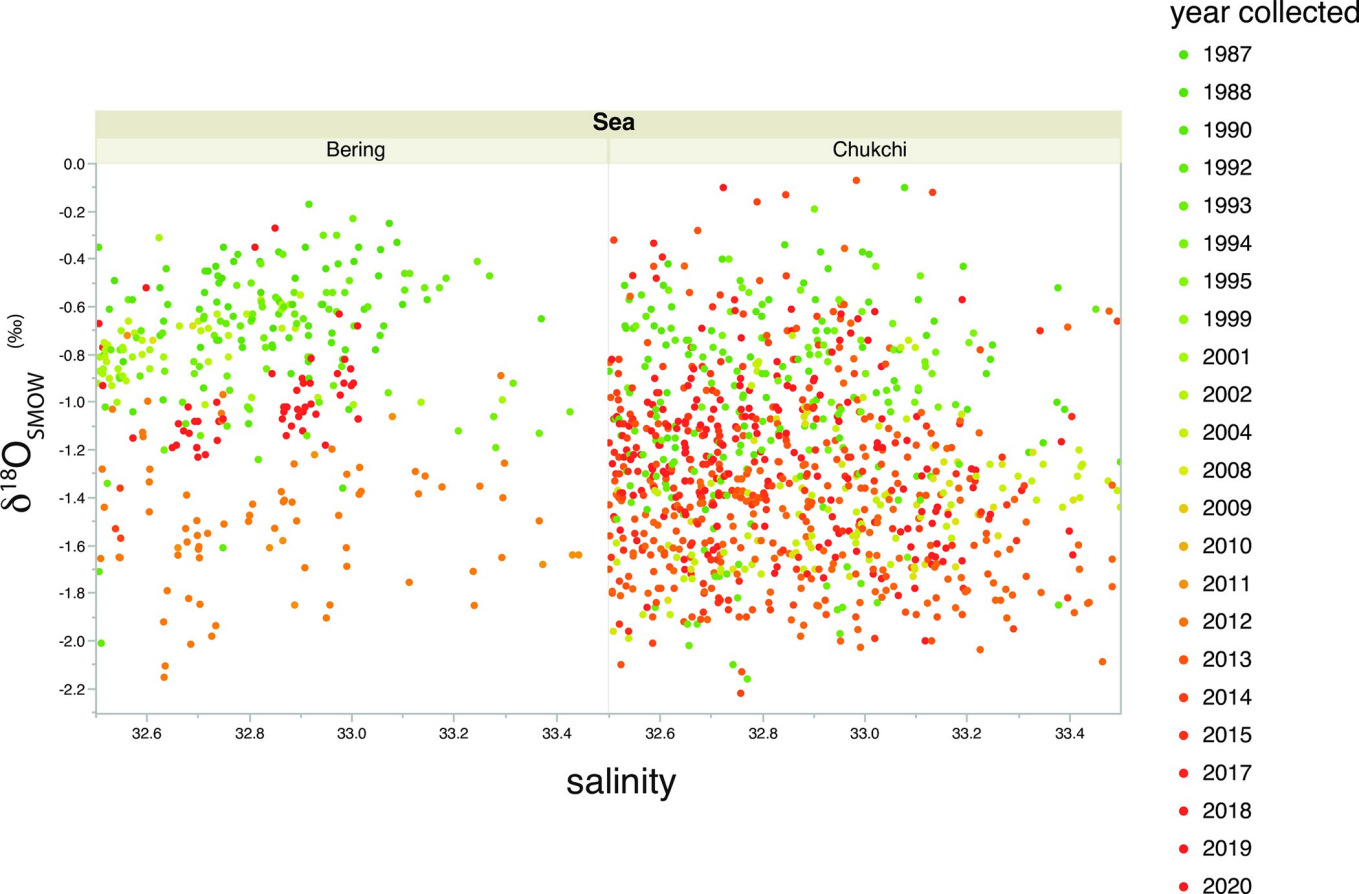
Distribution of δ^18^O values relative to salinity for 389 of the samples (Fig 3) collected in the Bering Sea, and 1016 samples (Fig 3) collected north of Bering Strait. Color coding of δ^18^O values by years follows that shown on Fig 3.

**Fig 5:**
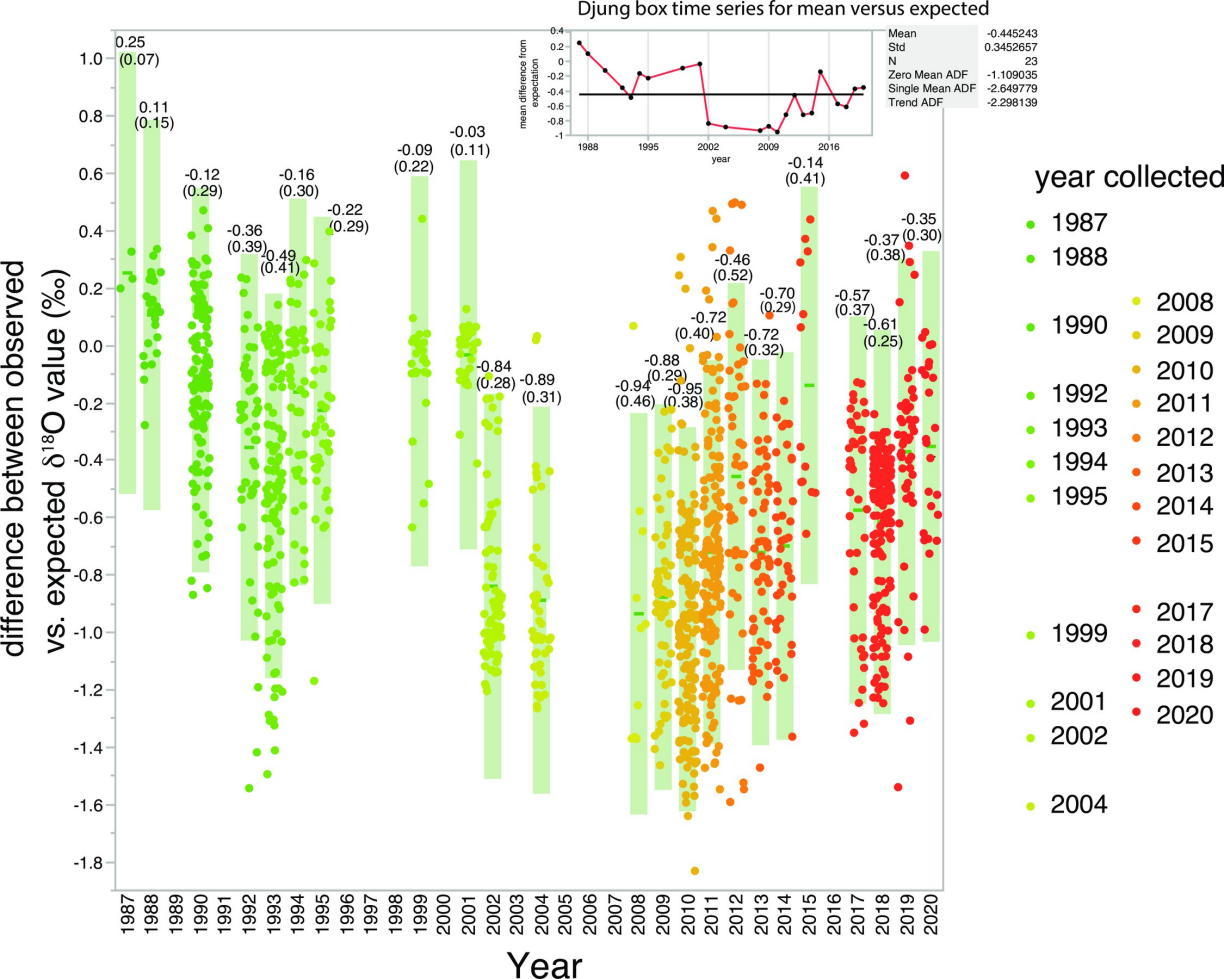
Data distribution by year with difference between expected δ^18^O value, based upon δ^18^O = Salinity * 0.59–20.0‰ (Eq 3; see text) versus the actual observed δ^18^O values for the observed salinities. Light shaded columns are confidence intervals associated with the mean deviation from the expected δ^18^O values. Numbers associated with each column are the mean deviation in observed samples from the expected δ^18^O values projected from Eq (3). Numbers in parenthesis are ±1 SD. Range of color coding of δ^18^O values by years as described previously in Fig 2.

A plot of salinity versus δ^18^O values show that there was a tendency for samples collected early in the time series (1987–2000) to be isotopically heavier than the idealized upper Arctic Ocean halocline (greenish points above horizontal line at -1.1‰; Fig 3). Qualitatively, in the years immediately following the turn of the century, water samples in this salinity range trended more negative and below the -1.1‰ threshold (yellowish points, Fig 3), but in the most recent years, for salinity of 32.5 to 33.5, there is a trend back towards the -1.1‰ threshold (reddish points). These patterns were apparent in both samples collected in the Bering as well as the Chukchi seas (Fig 4). In order to evaluate these apparent shifts in a more rigorous way, Eq (3) was used to predict the expected δ^18^O values based upon measured salinity and the difference between the expected and observed δ^18^O values were plotted by year (Fig 5). A Djung box time-series analysis (inset, Fig 5) indicated that the annual mean shifts were non-random and autocorrelated (p<0.05) except for 1995 (p = 0.07) and 1999 (p = 0.09). When analyzed for each year on a matched sample basis (paired t-test), the δ^18^O values observed were significantly different from the expected δ^18^O values predicted from salinity using Eq (3) for every year except 2001 and 2015 (Table 1). In addition, these differences remained significant whether samples were restricted to those collected north of Bering Strait, or included all samples regardless of location (Table 1).

**Table 1:** Matched pairs differences between observed δ^18^O values and predicted δ^18^O values from Eq (3), sorted by year. P is probability of the null hypothesis being true; there is no significant difference between the observed and expected δ^18^O values. P values and N (sample number) in italics are samples collected north of Bering Strait.

Observed δ^18^O values, mean (‰)	Expected δ^18^O values, mean (‰)	Mean difference (‰)	N (total)	N *(north of Bering Strait)*	P (all samples) *North of Bering Strait only*	year	Month of collection
-0.39	-0.64	0.25	3	*0*	0.02	1987	September
-0.51	-0.62	0.11	25	*1*	0.0014	1988	August-September
-0.74	-0.62	-0.12	111	*61*	<0.0001 *<0*.*001*	1990	June September
-1.04	-0.99	-0.35	51	*46*	<0.0001 *<0*.*0001*	1992	August
-1.14	-0.65	-0.49	109	*74*	<0.0001 *<0*.*0001*	1993	June August September-October
-0.74	-0.58	-0.16	50	*44*	0.0004 *<0*.*0001*	1994	May-June September
-0.80	-0.57	-0.23	44	*44*	*<0*.*001*	1995	September
-0.81	-0.72	-0.09	26	*0*	<0.045	1999	April
-0.74	-0.71	-0.03	31	*0*	<0.127	2001	March July
-1.39	-0.55	-0.84	79	*79*	*<0*.*0001*	2002	May-June August
-1.43	-0.54	-0.89	45	*43*	<0.0001 *<0*.*0001*	2004	May-June August
-1.46	-0.53	-0.93	10	*0*	<0.0001	2008	March
-1.50	-0.62	-0.88	69	*0*	<0.0001	2009	March
-1.52	-0.57	-0.95	143	*143*	*<0*.*0001*	2010	June-July
-1.35	-0.63	-0.72	162	*162*	*<0*.*0001* *<0*.*0001*	2011	July
-1.13	-0.68	-0.45	48	*45*	0.0212 *<0*.*0001*	2012	July August-September
-1.37	-0.65	-0.72	63	*63*	*<0*.*0001*	2013	August-September
-1.36	-0.67	-0.69	39	*39*	*<0*.*0001*	2014	August-September
-0.87	-0.73	-0.14	13	*13*	*0*.*2457*	2015	August-September
-1.26	-0.68	-0.57	43	*43*	*<0*.*0001*	2017	August-September
-1.27	-0.65	-0.61	163	*107*	<0.0001 *<0*.*0001*	2018	July August-September
-0.98	-0.61	-0.37	50	*29*	<0.0001 *<0*.*0001*	2019	July August-September
-0.95	-0.60	-0.35	24	*23*	*<0*.*0001*	2020	October

Interannual variation is apparent when the overall deviation in each year is considered. The mean variation for all years (n = 23) from the predicted δ^18^O values for the 23 years of sampling is -0.44 ± 0.36‰ SD. Location of sampling north or south of Bering Strait did not appear to be a critical variable. When the paired comparison between observed and expected δ^18^O values was separated between north and south of the Strait, in no year did the difference between observed and expected δ^18^O values change to significantly different or not significantly different (Table 1). In all years except 2001 and 2015, the difference between expected and observed δ^18^O values was significant (p<0.05).

## Discussion

These data show that the δ^18^O value of seawater in the North American Arctic with a potentially high enough salinity to reach the upper Arctic Ocean halocline following brine injection has varied interannually over a quarter century period. Based upon the assumption that the salinity of the upper Arctic Ocean halocline continues to be 33.1 and reflects Pacific-origin water flowing through the Bering Strait, we project that the change in the freshwater volume of water (in km^3^) passing through Bering Strait can be estimated by comparing:

$$\left(\delta^{18}O_{end‐member})(V_{init})=[(33.1)\;*\;(original\;\delta^{18}O\;value\;of\;upper\;halocline)\right]$$ (6)

relative to:

$$\left(\delta^{18}O_{end‐member})(V_{init}\pm V_{t})=[(33.1)\;*\;(new\;\delta^{18}O\;value\;of\;upper\;halocline)\right]$$ (7)

where V_init_ = freshwater volume in ~1990 near the start of the time-series, and V_t_ = change in freshwater volume in any year up until 2020. We assume in formulating these equations that the δ^18^O value of the freshwater end-member, meaning integrated snow and rain entrained in the Bering Strait inflow to the Arctic has not changed in composition over the time series of data (1987–2020) summarized here. For convenience, we can also use as a baseline the estimate of [1], 1670 km^3^, for the freshwater component (V_init_) of the Bering Strait inflow. The δ^18^O values used in these equations serve as a simplified form of the absolute isotopic ratios. As outlined in the introduction, the best fit for that freshwater end-member δ^18^O value within the upper halocline is a constant, -21.1‰, after considering the accepted oxygen isotope composition and salinity of the upper Arctic Ocean halocline (section 2.1). Likewise, we assume that the salinity of the upper Arctic Ocean halocline is constant at 33.1, and that it continues to correspond to the Bering Strait inflow, which is confirmed by the nutrient maximum associated with this salinity in the North American basins of the Arctic Ocean. Consequently, Eqs (6) and (7) can be simplified by factoring out the constants (freshwater end-member and salinity of the upper halocline) and solved for the remaining unknown, V_t_, which represents the change in volume of the freshwater flux through the Bering Strait from a late 1980’s baseline, 1670 km^3^:

$$V_{t}=[(\delta^{18}O_{init\;halocline})‐(\delta^{18}O_{t\;halocline}]/[(\delta^{18}O_{init\;halocline})*\;V_{init}],$$ (8)

where V_t_ is the freshwater flux at any time, t, δ^18^O _init halocline_ is the accepted 1980s δ^18^O value (-1.1‰) for water in the upper halocline (salinity of 33.1), (δ^18^O _t halocline_) is the oxygen isotope composition observed at any time, t, for water with that 33.1 salinity, and V_init_ is taken to be 1670 km^3^, the freshwater volume flowing through the Bering Strait in the 1980s [1].

Thus over the whole time-series (1987–2020), the mean deviation of -0.44‰ for all years (Fig 5) from the expected baseline (-1.1‰) δ^18^O value of the upper Arctic halocline indicates that a δ^18^O value of -1.5‰ is the actual longer-term mean. This corresponds to an average increase in the freshwater flux over the 23 years of sampling of 671 km^3^ from the estimated baseline of 1670 km^3^ in the 1980s or an increase of approximately 40%. Overall this estimate is similar to the increase in freshwater fluxes through the Bering Strait estimated from mooring measurements from 2001 to 2011, 40–50% [11]. While the years of the mooring record in the Bering Strait [2] and references therein do not exactly coincide with our record, the dates of the published record (1990–2015) are similar enough to merit comparison. One consideration of course is that we collected samples across both the Bering and Chukchi shelves (Table 1), so the spatial distribution of samples from the Chukchi Sea means that those water samples will be a number of months ahead of transport to the shelf break relative to those collected in the Bering Sea. Winter water samples collected in the Chukchi Sea may also be expected to have higher salinity for the same δ^18^O value, as brine is added on the Chukchi shelf. The interannual variation in the freshwater flux through Bering Strait was reported to be 1200 km^3^ [2], which is somewhat smaller than the interannual range estimated by Eq (7), 1611 km^3^, ranging from 1507 km^3^ in 1988 (we exclude 1987 based upon limited data) to 3118 km^3^ in 2010 (mean = 2341 km^3^ ± 545 s.d.); these estimates are consistent with a high degree of interannual variability for freshwater fluxes that are observed in the mooring data.

**Fig 6:**
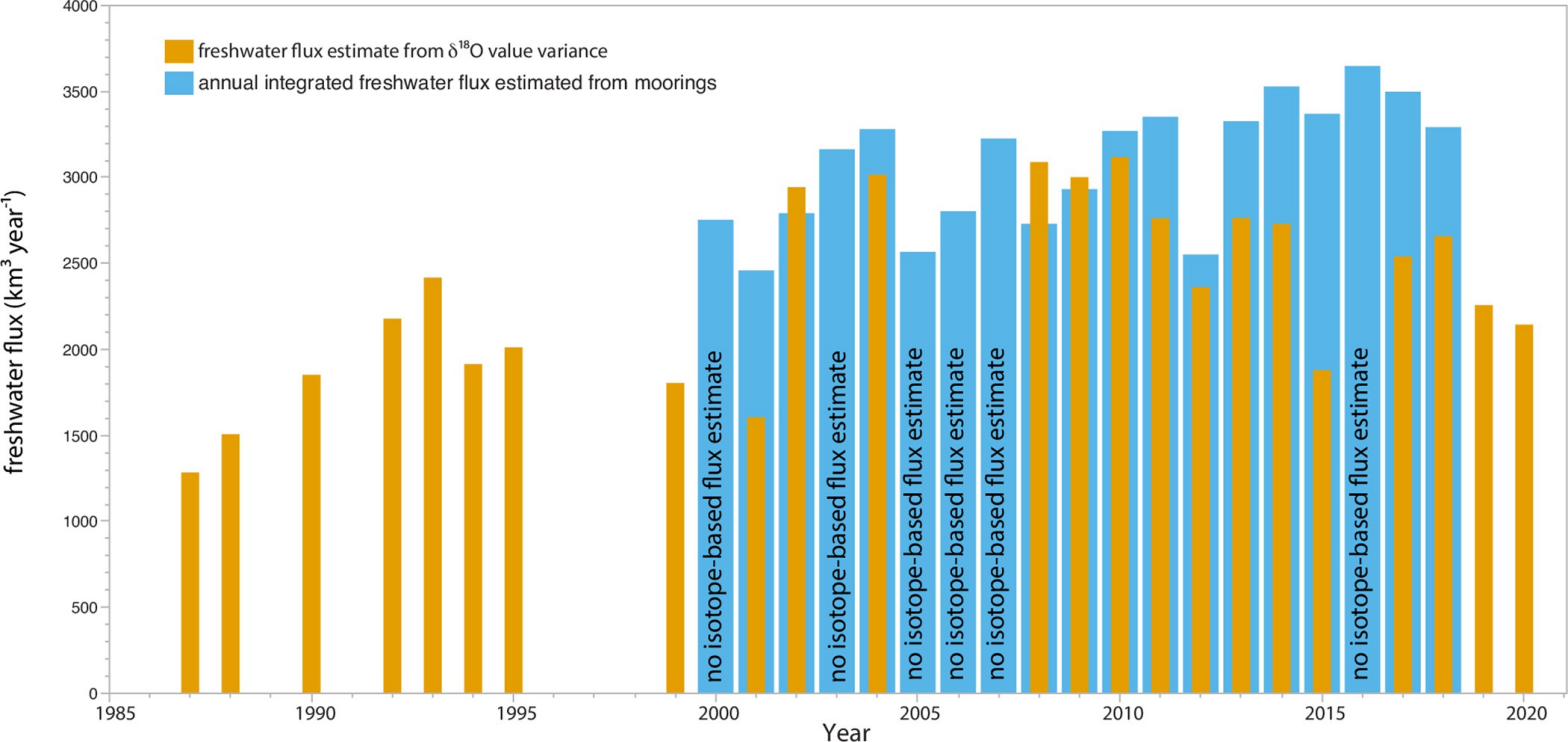
Estimated freshwater flux through Bering Strait using oxygen isotope variation versus mooring estimates (data from [2]).

The data from mooring site A4, in Alaska Coastal Water in the eastern Bering Strait, together with site A3, north of the Strait (Fig 1, inset), but within the center of flow, are used as the basis for estimating integrated annual freshwater transport through the Bering Strait, relative to a deep Arctic Ocean salinity of 34.8 [2,24]. We can compare the estimates of the freshwater flux from these moorings on a year-to-year basis with the estimates based on the isotopic variation observed. We do this using the baseline estimate of the freshwater flux from the late 1980’s (1670 km^3^; [1] and then apply Eq 8 to estimate the change in the freshwater flux from that assumed baseline. The computed freshwater fluxes are compared to those from [2] and show a similar, significant increase in the freshwater flux after the turn of the century. However, the isotope-derived estimate declines somewhat after 2010 while the mooring based estimate remains high within the past decade (Fig 6). While some years show very good agreement between the two estimates, e.g. 2002, 2004, 2008, 2009, 2010, and 2012, overall, the two year by year estimates are not significantly correlated (Pearson product moment correlation, p = 0.42). One likely reason is that the timing of water sample collection for stable isotopes is not exactly aligned in time and space with the mooring measurements and it is different each year, depending upon ship track and timing. Because of seasonal ice cover extent, many of the oxygen isotope determinations are from samples predominantly collected during late summer, in some cases many months after the water measured at the moorings has passed through the Bering Strait. However, in the case of the samples collected in 2002, 2004, 2008, 2009, and 2010 (but not 2001), when there was good agreement (Fig 6), sampling occurred during early spring cruises in support of the Shelf-Basin Interactions [19] and Bering Sea Programs [12]. In these cases, sampling was as early as March, as well as during April-June while there was active brine injection underway. This suggests that use of oxygen isotope variability to estimate freshwater transport in the Bering Strait may have the best fidelity to same-year mooring based estimates early in the seasonal cycle when brine injection is still underway and dense water reaching the upper halocline is possible. However additional sampling is necessary to verify this initial indication.

Another consideration in estimating these changes in freshwater fluxes is that it means that there has been a significant change in the mean salinity of waters transiting the Bering Strait. However, the mooring array is fixed and the ship-based measurements we have made vary in time and place. Since the water masses in the Strait respond strongly to changes in wind forcing, e.g. [18], it is actually most practical to estimate those changes from Eq (3), which describes the relationship between δ^18^O and salinity for waters present in the Bering Strait, before brine injection occurs on the Chukchi shelf. Given the mean change per year between 1987 and 2020 of -0.44‰ in δ^18^O values, an overall salinity decrease of 0.68 would be required to match that mean decrease in δ^18^O values over the 23 years in sampling, based upon the use of Eq (3), which applies to waters in the vicinity of Bering Strait. This degree of reduction in salinity is consistent with the recent mooring observations [24,28], which have been interpreted to indicate that high nutrient waters passing through Bering Strait are no longer saline enough to reach a salinity of 33.1, which could possibly explain the lack of coherence in the past decade between the isotope-based estimates of freshwater fluxes (which depend upon a constant 33.1 salinity for the Bering Strait inflow) and those fluxes estimated by the mooring measurements. The assumption we have made that the 33.1 salinity for the core of Bering Sea winter water has not varied would in that case no longer be true. Clearly despite the extensive data set available, full reconciliation of the mooring based measurements and isotope-based estimates will require additional sample collection if it is true that winter water flowing through Bering Strait no longer reaches a salinity of 33.1.

## Conclusions

A large oxygen isotope data set was used to estimate freshwater fluxes to the Arctic Ocean through Bering Strait, following empirical criteria about the salinity of samples likely to contribute to the upper Arctic halocline. These estimates of freshwater fluxes do not perfectly align with the mooring record from the Bering Strait, but the overall patterns and scale of increasing freshwater fluxes into the Arctic in the past several decades are consistent in both approaches. Sampling that was predominantly under late winter or spring, ice-covered conditions seemed to provide freshwater flux estimates that were better correlated with mooring estimates, so additional sampling at appropriate times and salinities may be required to refine empirically the best criteria for using stable oxygen isotope composition to evaluate the changing freshwater fluxes contributed to the Arctic Ocean via the Bering Strait.
